# Ultrasonic Characterization of the Fiber-Matrix Interfacial Bond in Aerospace Composites

**DOI:** 10.1155/2013/154984

**Published:** 2013-06-27

**Authors:** D. G. Aggelis, D. Kleitsa, T. E. Matikas

**Affiliations:** ^1^Department of Mechanics of Materials and Constructions, Vrije Universiteit Brussel, Pleinlaan 2, 1050 Brussels, Belgium; ^2^Department of Materials Science and Engineering, University of Ioannina, 45110 Ioannina, Greece

## Abstract

The properties of advanced composites rely on the quality of the fiber-matrix bonding. Service-induced damage results in deterioration of bonding quality, seriously compromising the load-bearing capacity of the structure. While traditional methods to assess bonding are destructive, herein a nondestructive methodology based on shear wave reflection is numerically investigated. Reflection relies on the bonding quality and results in discernable changes in the received waveform. The key element is the “interphase” model material with varying stiffness. The study is an example of how computational methods enhance the understanding of delicate features concerning the nondestructive evaluation of materials used in advanced structures.

## 1. Introduction

**Figure 1:**
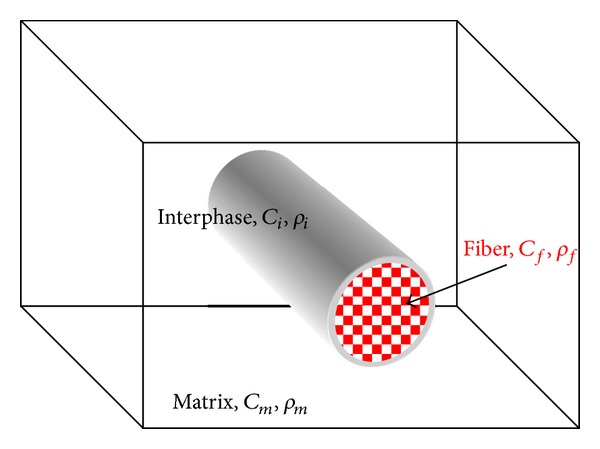
A typical part of the microstructure of the composite.

Reinforcement of a bulk material with fibers is commonly applied in order to upgrade its properties in terms of stiffness, strength, and durability. Fiber composites are applicable in any type of material, like steel-fiber-reinforced concrete, polymer, or ceramic composites as well as metal matrix composite materials [1–3]. In most cases, the fibers exhibit higher mechanical properties than the matrix to improve its behavior. However, in order to take full advantage of the fiber potential, the bonding between fiber and matrix is of primary importance. Efficient stress transfer is desirable, and this is the reason that in certain cases chemical treatment of the fibers is applied in order to enhance bonding [4]. The chemical reaction between the matrix and fiber results in an “interphase” zone with properties different than the ones of the constituent phases (see Figure 1). This interphase zone may be very thin but it plays a crucial role in the mechanical performance of the medium. This is the zone through which stress is transferred, and therefore, all important mechanical properties of the composite like its strength and toughness heavily depend on the quality of the interphase [5, 6]. However, environmental and stress effects degrade the quality of the interphase compromising the structural capacity of the whole composite. It is understandable that the initial bonding conditions between fiber and matrix should be optimized in order to minimize the effect of service-induced deterioration. Assessing the quality of initial bonding is a task that can be conducted by certain mechanical tests like pull-out or push-in [7, 8]. However, these are destructive and focus on the strength, totally neglecting the elastic properties of the interphase. Therefore, a fast, nondestructive, and simple methodology of assessing the quality of the interphase is desirable. This should result in a quantifiable parameter related to the stiffness of the interphase and could be applied after manufacturing to assess the initial bonding condition in single fiber specimens or, under suitable circumstances, after service for assessment of bonding degradation. A reliable test will assist the design of the composite material by evaluating the stiffness of the interphase in terms of the different constituent materials' elastic and thermal properties, as well as regarding the suitable conditions to achieve optimal bonding, that is, temperature and pressure.

In the present paper, a numerical study of stress wave reflection is described in order to estimate the potential for characterization of the bonding and guide relevant experimental efforts [9]. The suitability of stress waves has been pointed out concerning characterization of surfaces and thin layers [10]. Stress waves employ infinitesimally small displacements and are influenced by the elastic properties of the materials. Therefore, their study reveals information on the stiffness of the materials and not directly their strength. Particularly, the immersion pulser-receiver technique is targeted. According to this technique, a pressure pulse is emitted inside a liquid (normally water). This pulse propagates with the sound velocity of water towards the material under test. When it meets an interface, it is partially transmitted and partially reflected through the medium. The number of reflections recorded by the sensor and their delay define the possible defects and their depth below the surface of the tested object. In the specific case described herein, the direction of the pulse relatively to the materials' surface is dictated by the shear critical angle so that only shear waves propagate in the medium, which are arguably more sensitive to the existence and quality of interphases [8, 11, 12]. The shear wave interacts with the embedded fiber and the reflection is eventually recorded by the receiver. Analysis of the recorded waveform sheds light into the condition of the interphase since the reflection depends on the relative mismatch of mechanical properties. When the bonding is inadequate due to incompatible materials or has worn out, there is essentially no contact between the materials. Therefore, a strong reflection is bound to occur since the fiber volume acts as a void in terms of wave propagation. In the case of a single scatterer, although dispersive effects are not expected, the analysis is based on the reflection on the scatterer in a pulser-receiver mode. On the other hand when two materials of similar stiffness are in good contact, the reflection will be minimized. The different possible conditions of bonding are simulated herein by an elastic “interphase” material with varying stiffness. This interphase should not be confused with the “interface” which is a boundary between the matrix and the fiber. This interphase is used to model the elastic properties of the interphasial zone between the matrix and fibers resulting from the chemical reaction between the two materials. Hence its behavior is governed by a user-defined varying elastic wave velocity to simulate different degrees of stiffness. This study includes the exact geometry of the fiber as an advancement of the analytical solution that was provided in [9] for the reflection on an inclined straight line instead of the circular cross-section of the fiber. The material system targeted herein is a metal matrix composite material [9]. Specifically, the matrix of the targeted material is Ti-6Al-4V reinforced with continuous SCS-6 fibers, a material widely used in aerospace. High strength titanium alloys, as well as fiber-reinforced metal matrix composite materials, are suitable for a number of highly demanding applications because of their improved mechanical properties in high temperature conditions. In applications where dynamic loading is expected and where life management is required, consideration must be given to the behavior of the material in the sensitive area of the interphase between the matrix and the fiber in order to verify the best possible performance of the material.

## 2. Numerical Simulations

### 2.1. Model

Numerical simulations are generally used to expand to cases that cannot be experimentally tested due to cost, geometry, or other limitations and also to increase the physical understanding in specific problems. Wave simulation studies enable also the recognition of wave modes and reflections inside a whole waveform. In the specific case, two-dimensional simulations were conducted on a cross-section of the geometry as is explained below. 

The fundamental equation of two-dimensional propagation of elastic waves in an elastic medium neglecting viscosity is

(1) $$\begin{array}{c} \rho \frac{\partial^{2}\underline{u}}{\partial t^{2}}=\mu \nabla^{2}\underline{u}+\left(\lambda +\mu \right)\nabla \nabla \cdot \underline{u}, \end{array}$$

where $\underline{u}=u(x,y,t)$ is the displacement vector as a function of time,*t*, *ρ* is the material density, and *λ* and *μ* are the first and second Lame constants, respectively. These parameters are related to the wave propagation velocities with the following equations:

(2) $$\begin{array}{c} C_{L}=\sqrt{\frac{\lambda +2\mu }{\rho }},\\ C_{S}=\sqrt{\frac{\mu }{\rho }}, \end{array}$$

where *C*_*L*_ is the longitudinal and *C*_*S*_ is the shear wave velocities respectively.

The simulations were conducted with commercially available software [13] that solves the above equation with respect to the boundary conditions of the object and the initial conditions [14]. The solution is in time domain with the finite difference method in the plane strain field. The excitation pulse has a defined displacement-time function and is applied at specified nodes of the geometry that simulate the “pulser.” Continuity equations must be fulfilled at the interfaces between different entities. In the present analysis, individual materials are included in the geometry, and therefore propagation is solved in each distinct phase, while the continuity conditions for stresses and strains must be satisfied on the interfaces. 

**Figure 2:**
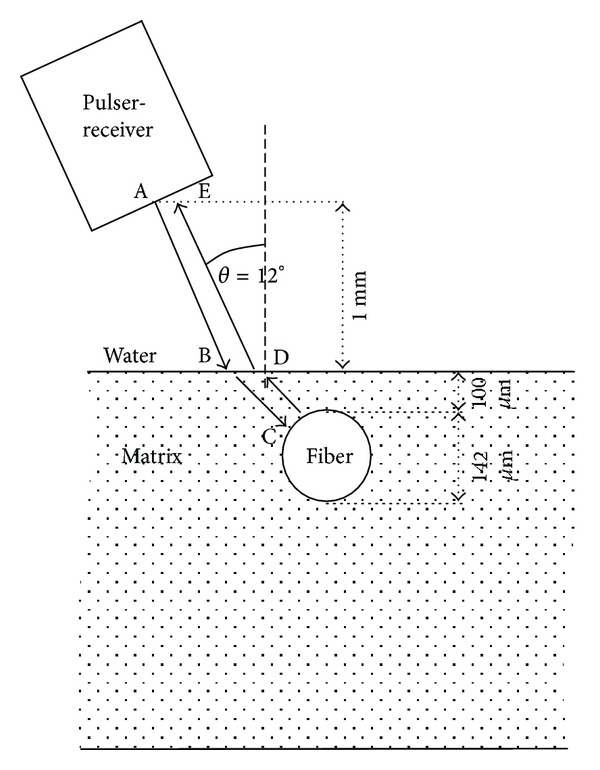
Geometry of the simulated test including wave directions.

In the case described herein, the propagation of a stress wave after excitation in water is simulated. The geometry is shown in Figure 2 and the wave path of interest is indicated, while it is discussed in more detail in Section 3. The wave impinges on the matrix under the shear critical angle, thus allowing only shear waves to propagate into the matrix. The shear wave interacts with the fiber and a part is reflected back. After being refracted from the matrix/water interface, a longitudinal wave propagates through water back to the receiver (same as pulser, see Figure 2). 

The “source” is placed at a specific angle, *θ* relatively to the vertical axis, equal to the critical shear angle of this horizontal liquid/solid interface. In the specific case, the angle is 12°, as calculated based on Snell's law and the mechanical properties of water and the titanium matrix [9]. The pulser introduces one cycle of different frequencies in the longitudinal mode. The applied frequencies were 1 MHz, 5 MHz, 10 MHz, 25 MHz, and 50 MHz. 

The employed materials were considered elastic without viscosity. The basic properties of all the materials except the interphase are seen in Table 1. Both matrix and fiber materials are quite stiff with the fiber exhibiting approximately twice the longitudinal and shear wave velocities of the matrix. As already mentioned, the interphase obtained different values of stiffness expressed by the corresponding longitudinal wave velocities. This is a key parameter of the study and a practical way to simulate different contact levels between the matrix material and the fiber [11, 15]. Specifically, the lowest value was 300 m/s (case of loose interphase similar to air), and the maximum 11770 m/s which is the longitudinal wave velocity of the fiber. In between, the values were incremented by 1000 m/s, for example, 1000 m/s, 2000 m/s, 3000 m/s, and so forth. This includes the possible range of equivalent stiffness values that could be obtained by the interphase layer. The diameter of the fiber is 142 *μ*m, and it is embedded 100 *μ*m below the surface, (see Figure 2). Since there was no physical insight for the thickness of the actual interphase layer, it was set to 50 *μ*m. In similar cases, it has been shown that the thickness of the interphase does not make critical difference in the results [11]. The vertical distance of the pulser was indicatively set to 1 mm above the surface of the specimen, while it can be adjusted to suit the relevant experimental geometry each time. 

**Figure 3:**
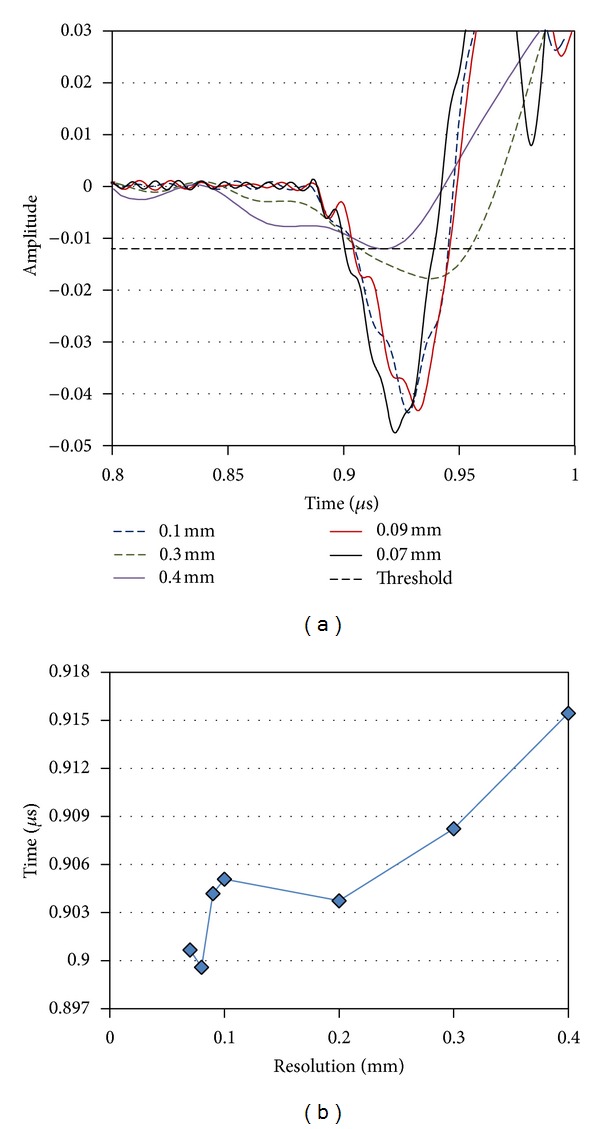
(a) Onset of the reflection in the received waveform for different resolutions. (b) Arrival time to the receiver for different resolutions (measured from the threshold crossing at 0.012 u.a.).

As in any simulation study, here also certain conditions must apply in order to ensure reliable and repeatable results. The mesh size is a crucial parameter since if it is defined to a relatively large value, the outcome will not be accurate but on the other hand there are computational power and time restrictions that prevent from applying an infinitesimally small value. Restrictions on the computational power do not always allow to use several elements per wavelength. In any case, since the study employs four materials (water, matrix, fiber, and interphase), there is no standard wavelength to adjust the element size accordingly. Therefore, another holistic approach was followed; different values of mesh sizes were tested; namely, from 0.4 mm down to 70 *μ*m and the resulted waveforms were compared.  Figure 3(a) shows the time window when the first part of the reflection (case of a loose interphase) is recorded for the frequency of 25 MHz for some indicative mesh sizes. Simulations with mesh sizes larger than 0.1 mm (specifically 0.3 and 0.4 mm in Figure 3(a)) result in quite different waveforms compared to the finer meshes and were not further considered. From the mesh size of 100 *μ*m and finer, the waveforms converge in shape. In order to quantify the comparison of these cases, a threshold was chosen, namely, −0.012 units of amplitude (u.a.) in order to deterministically define the onset of the reflection (see Figure 3(a) and compare between different cases). As the mesh becomes finer, the calculated onset times changed and can be seen in Figure 3(b). The finer mesh tested (70 *μ*m) resulted in an onset of 0.90067 *μ*s but it was extremely time consuming. The simulations were conducted with the mesh of 80 *μ*m which resulted in transit time of 0.89959 *μ*s being 0.12% away from the result of the finest mesh applied. This was considered a suitable approximation due to the limited amount of error relatively to the specific available computational power. As an indication, a full simulation of a case in a computer with RAM of 3 GB and processor of 2.1 GHz lasted about 1 hr. Concerning the time step resolution, it resulted in 0.00034 *μ*s, which even for the highest frequency (50 MHz with period of 0.02 *μ*s) contains approximately 55–60 points in a cycle and is considered more than adequate sampling in similar cases [16]. 

## 3. Results

**Figure 4:**
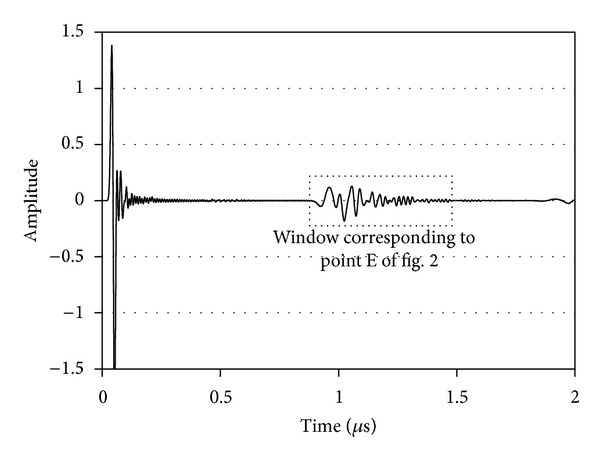
Typical waveform after excitation of 25 MHz.

The longitudinal wave pulse is emitted by the pulser (point A in Figure 2). This pulse propagates initially through water and hits the water/matrix interface under the shear angle, *θ*, as has been discussed above (point B). The shear wave is transmitted through the matrix and reaches the fiber (C). Reasonably one part is reflected and another is transmitted past the fiber. The amount of energy reflected will depend on the shear wave impedance (product of shear velocity and density) mismatch of the two materials. The matrix impedance is of the level of 9 MRayl, while the fiber which is stiffer exhibits impedance of 23 MRayl. Therefore, in any case a reflection is expected when the materials are in perfect contact. If, on the contrary, the fiber is totally debonded from the matrix, the reflection will be stronger since the impedance of air is negligible compared to that of the matrix. It is reasonable that for any intermediate condition of bonding quality the reflection will be in between the above-mentioned extreme cases. This role (quality of bonding) is played by the “interphase” material, which in our analysis obtains variable values of stiffness, as expressed by the different longitudinal wave velocities. The wave reflected by the fiber, which now may again include longitudinal components after the reflection on the circular surface, propagates back to the surface of the matrix (D), and a part is refracted within water as longitudinal wave following the opposite direction of the initial incident pulse. This wave reaches the sensor as shown in Figure 2, point E. A typical waveform is seen in Figure 4 where the initial pulse and the reflection (window corresponding to point E of Figure 2) are shown, and in this part of the wave any analysis and evaluation should be focused to characterize the quality of the interphase.

**Figure 5:**
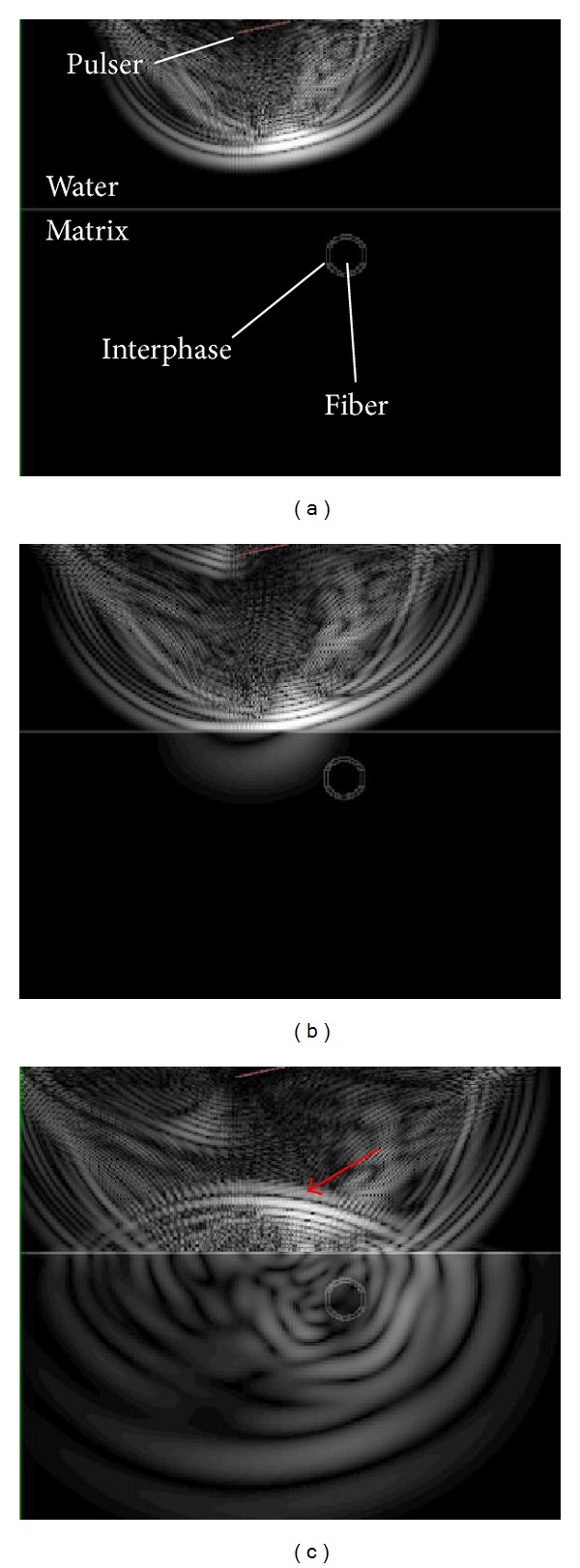
Consecutive snapshots of the displacement field for the case of stiff interphase.

**Figure 6:**
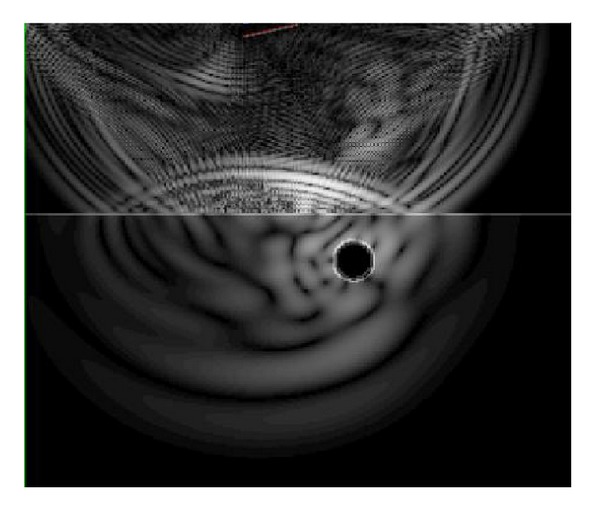
Snapshot of the displacement field for the case of loose interphase.

Figure 5 shows some indicative views of the displacement field for the frequency of 25 MHz and for the stiff interphase with pulse velocity of 11770 m/s. In the first case (a), the wave is propagating through water, while in Figure 5(b) the shear wave starts to be refracted in the matrix traveling on a higher speed than the wave in water. In the last case of Figure 5(c), the clear reflection can be seen in water (see arrow) while the refracted wave propagates deep in the matrix.  Figure 6 shows the field at approximately the same time but with loose interphase. It is obvious that no wave is transmitted through the fiber, while the reflection traveling back to the receiver is similar to the previous case. However, it contains critical differences that make characterization of the different interphases possible, as discussed next.

**Figure 7:**
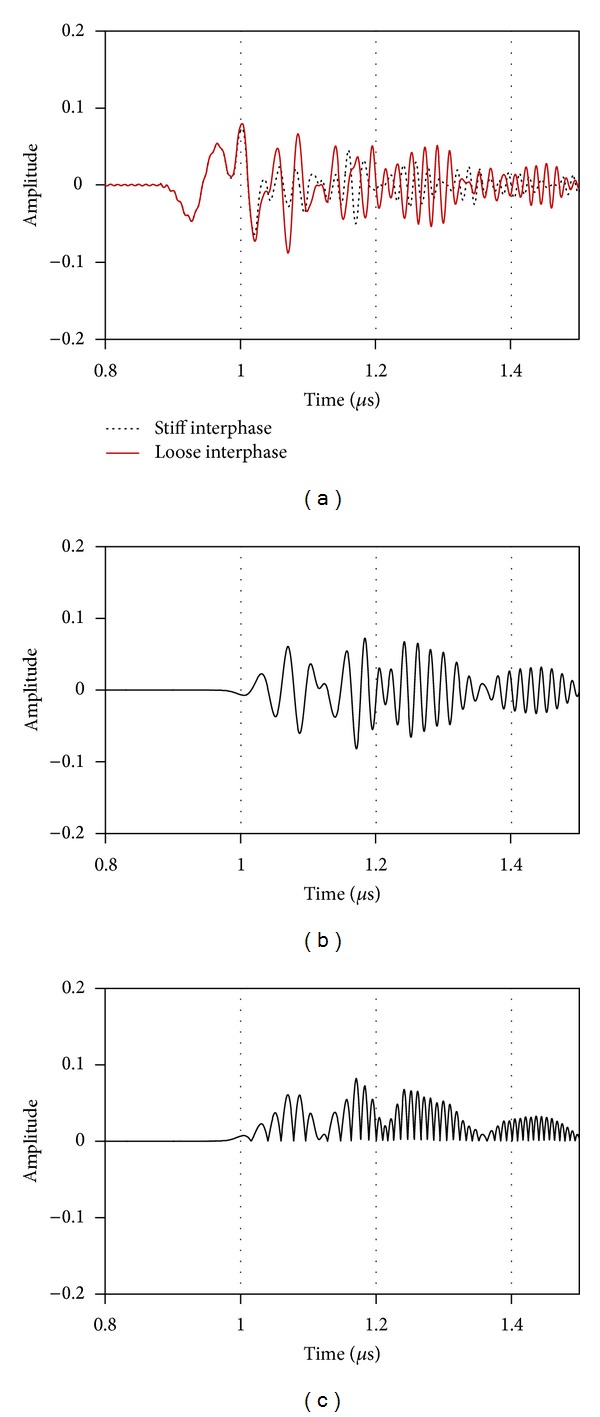
(a) Part of the waveform containing the reflection from the fiber for stiff (*C*_*i*_ = 11770 m/s) and loose (*C*_*i*_ = 300 m/s) interphase. (b) Subtraction of the two waveforms of (a). (c) Rectification of the waveform of (b).

**Figure 8:**
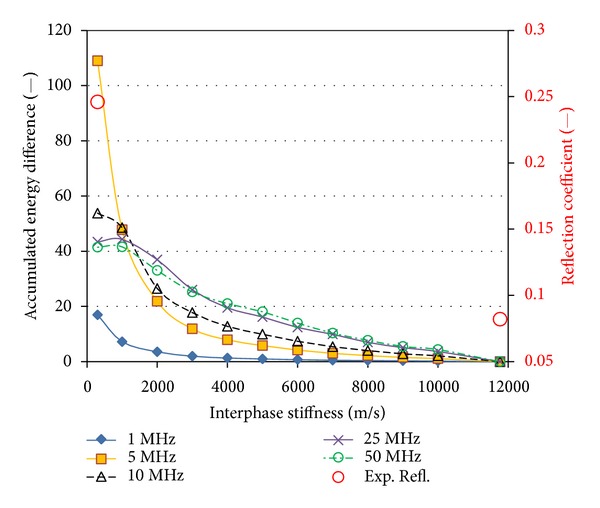
Reflection energy difference for several values of interphase stiffness (longitudinal velocities) and different frequencies.

Figure 7(a) shows the reflections (corresponding to window E of Figure 4) as recorded by the receiver for two extreme cases of interphase stiffness values, namely, equivalent to air (*C*_*i*_ = 330 m/s) and fiber (*C*_*i*_ = 11770 m/s). The waveforms are identical up to 1 *μ*s, since the initial part of the waveform is due to the direct reflection on the water/solid interface which is not influenced by the fiber. The wave packet of the reflection between the matrix/fiber interphase arrives slightly later since the fiber is at a depth of 100 *μ*m from the surface. Therefore, some discrepancies are visible after the time of 1 *μ*s, with the waveform from the loose interphase exhibiting higher amplitude attributed more likely to the higher reflection coefficient. In order to focus on the differences between the two waveforms, they are subtracted and the resulted waveform is seen in Figure 7(b). Quite detectable discrepancies are noted after 1 *μ*s. The result of the subtraction is a wave of similar amplitude mainly because the reflections from a less stiff second material are of opposite phase. The discrepancy can be quantified by the area of the signal envelope (measured area under the rectified signal envelope, see Figure 7(c)) denoted as “energy,” which is a parameter widely used in waveforms analysis [17, 18]. The reflection from the stiff interphase was maintained as reference and the waveforms obtained for each other stiffness were subtracted by the reference in order to calculate the energy difference. The results are seen in Figure 8. For any of the applied frequencies, this energy indicator increases monotonically as the interphase stiffness decreases from its maximum value down to the value of loose interphase. This is because the reflection from the fiber with a loose interphase is maximum due to the extreme impedance mismatch, as has already been mentioned. Comparing the results derived for different frequencies, the maximum energy difference comes for the frequency of 5 MHz, where its value is more than 100 units, while its lowest for loose interphase comes at 1 MHz. Frequencies of 10, 25, and 50 MHz result in intermediate values of 40 to 55, while specifically 50 MHz exhibits a quite constant rate, being equally sensitive to changes of interphase velocity at any interphase velocity level. On the contrary, the 5 MHz curve is very sensitive to changes at the low level of interphase velocities but is not as sensitive to higher values close to good bonding (i.e., the initial signs of debonding in a real case). Therefore, in actual application the use of higher frequencies (25 MHz or 50 MHz) is suggested for assessment of even slight incompatibility or debonding trends, which corresponds to a drop of interphasial stiffness from 11770 m/s to 10000 m/s or 15%. In the same figure, the experimental values of the reflection coefficient for the two extreme cases (good bonding and simulated debonding) are also included, as measured in [9]. This reflection coefficient was obtained by comparing the FFT of the waveform corresponding to the actual geometry (e.g., with a hole) to the waveform of an angled surface which was considered a reference. This reflection coefficient, which again depends on the mismatch between the two sides of the interface, is much higher for the debonding than the case of regular bond between the fiber and the matrix. The qualitative similarity in the decreasing trend between the experimental and numerical energy-related features as the interphasial stiffness increases shows that the approach is in the right direction, and further study will enable accurate evaluations of the interphase quality.

## 4. Discussion 

The results presented above show that ultrasonic reflection parameters exhibit a monotonic trend with respect to the interphase stiffness. This opens the possibilities not only to detect debonding or poor compatibility but to quantify the stiffness of the interphase. This property is handled by the equivalent wave velocity of the modeling material called “interphase” in this study in accordance to the actual layer between the matrix and fiber materials. The values of interphase stiffness are varied from the two extreme cases of similar to air (loose contact) and similar to fiber (strong bonding) including all the possible realistic values in between. Concerning some specific parameters that are encountered towards the experimental application, it should be mentioned that though the measurement is delicate, in a real experiment with the immersion technique, the quality of the acoustic coupling provided by water is constant and therefore, any difference due to even slight reflection changes will be detected. The sensor scans along the longitudinal axis of the fiber enabling characterization of the interphase bonding on its whole length. It should be kept in mind that this test is intended for material design purposes (compatibility of constituents) rather than deterioration assessment. Therefore, the targeted geometry is simple (e.g., single fiber specimen [9]), in order to avoid the interference with neighboring fibers that would occur in the actual material. The simple geometry will enable derivation of accurate information on the fiber-matrix interphase and will act as a guide for the material design process. This way the results from different systems can be compared in order to judge sort their interface compatibility. Additionally, the corresponding “stiffness” of the interphase can be correlated to the results of mechanical tests if they are also performed (i.e., pull-out or push-in). Concerning the fiber alignment, which is crucial for the aforementioned destructive tests, it is not crucial for the proposed ultrasonic reflection technique because the experimental wave beam cross-section is much larger than the fiber diameter. 

## 5. Conclusion 

Advanced metal matrix composites for aerospace applications require delicate methods to accurately assess their initial state as well as service-induced damage. This study concerns the nondestructive evaluation of the quality of bonding between fiber and matrix in such composites. The exact fiber geometry is simulated as an advancement of the previous analytic studies on a simplified geometry. The immersion ultrasonic technique is numerically simulated, while shear waves are targeted due to their sensitivity on bonding conditions. Different bonding is modeled by altering the stiffness of the “interphase” material which acquires properties from near-zero, simulating negligible contact up to stiffness similar to the fiber, simulating the stiffest possible bonding. The results indicate that despite the consecutive refractions between the water and matrix, the influence of the de-bonding is distinguishable compared to the case of stiff interphase. This is because the amount of energy reflected depends on the interphase elastic properties which cause small but discernible differences in the received waveform. The study shows how computational methods enhance our understanding and can give direction to the relevant experimental techniques with the aim of providing better characterization of crucial aspects of the material's condition in a nondestructive manner. 

## Figures and Tables

**Table 1:** Basic properties for material modeling.

	Water	Matrix (Ti-6Al-4V)	Fiber (SCS-6)
*λ* (GPa)	2.25	25.9	61.9
*μ* (GPa)	10^−4^	32.8	177.0
*ρ* (kg/m^3^)	1000	2580	3000
*C*_*L*_ (m/s)	1500	5954	11774
*C*_*S*_ (m/s)	10	3566	7681
